# Contribution of biotransformation enzymes to the development of renal injury and urothelial cancer caused by aristolochic acid: urgent questions, difficult answers

**DOI:** 10.2478/v10102-010-0023-1

**Published:** 2010-11

**Authors:** Marie Stiborová, Jiří Hudeček, Eva Frei, Heinz H. Schmeiser

**Affiliations:** 1 Department of Biochemistry, Faculty of Science, Charles University, 128 40 Prague 2, Czech Republic; 2 Division of Molecular Toxicology, German Cancer Research Center, 69120 Heidelberg, Germany

**Keywords:** Aristolochic acid, metabolism, Aristolochic acid- and Balkan endemic-nephropathy, renal injury, tumor induction

## Abstract

Ingestion of aristolochic acid (AA) is associated with the development of aristolochic acid nephropathy, which is characterized by chronic renal failure, tubulointerstitial fibrosis and urothelial cancer. AA may also cause a similar type of kidney fibrosis with malignant transformation of the urothelium, the Balkan endemic nephropathy. Understanding which enzymes are involved in AA activation and/or detoxication is important in the assessment of a susceptibility to this carcinogen. The most important human enzymes activating AA by simple nitroreduction *in vitro* are hepatic and renal cytosolic NAD(P)H:quinone oxidoreductase, hepatic microsomal cytochrome P450 1A2 and renal microsomal NADPH:cytcohrome P450 reductase, besides cyclooxygenase, which is highly expressed in urothelial tissue. Despite extensive research, contribution of most of these enzymes to the development of these diseases is still unknown. Hepatic cytochromes P450 were found to detoxicate AA in mice, and thereby protect the kidney from injury. However, which of cytochromes P450 are the most important in this process both in animal models and in humans have not been entirely resolved as yet. In addition, the relative contribution of enzymes found to activate AA to species responsible for induction of urothelial cancer in humans remains still to be resolved.

## Introduction

**Figure 1:**
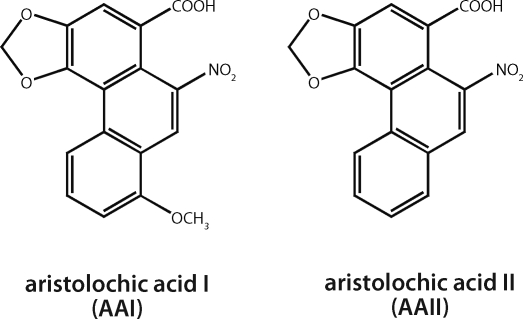
Aristolochic acid I and II.

Aristolochic acid (AA), the plant extract of *Aristolochia* species, is a mixture of structurally related nitrophenanthrene carboxylic acids, with 8-methoxy-6-nitro-phenanthro-(3,4-*d*)-1,3-dioxolo-5-carboxylic acid (AAI) and 6-nitro-phenanthro-(3,4-*d*)-1,3-dioxolo-5-carboxylic acid (AAII), being the major components (Figure 1) (IARC, 2002). Recently AA was proven to be the cause of so-called Chinese herbs nephropathy (CHN), a unique type of rapidly progressive renal fibrosis associated with the prolonged intake of Chinese herbal remedies during a slimming regimen, observed for the first time in Belgium in 1991 (Vanherweghem *et al*., 1993; Vanhaelen *et al*., 1994; Schmeiser *et al*., 1996). About 100 CHN cases have been identified so far in Belgium, half of which needed renal replacement therapy, mostly including renal transplantation (Arlt *et al*., 2002b). The observed nephrotoxicity has been traced to the ingestion of herbal preparation *Aristolochia fangchi* containing nephrotoxic AA inadvertently included in slimming pills (Vanhaelen *et al*., 1994). CHN patients, who were exposed to *Aristolochia* species containing AA and had no relationship with the Belgian slimming clinic, have been identified in other European countries, in Asia and in the USA (about 170 cases) (Arlt *et al*., 2002b). Therefore, this disease is now called aristolochic acid nephropathy (AAN) (Gillerot *et al*., 2001; Arlt *et al*., 2002b; Cosyns, 2003). Recently, a high prevalence of urothelial cancer was found in the cohort of AAN patients in Belgium (Nortier *et al*., 2000) and cases of urothelial cancer have also been described in other countries (Arlt *et al*., 2004). These findings highlight the carcinogenic potential of AA to humans. Indeed, AA is among the most potent 2% of known carcinogens (Arlt *et al*., 2002b; IARC, 2002). As a consequence, herbal remedies containing species of the genus *Aristolochia* were recently classified as carcinogenic to humans (Group 1) by the International Agency for Research on Cancer (IARC) (IARC, 2002).

It is also noteworthy that AA consumption may be a cause for the development of a similar type of kidney fibrosis with malignant transformation of the urothelium, the Balkan endemic nephropathy (BEN) (Ivic, 1969; Tatu *et al*., 1998; Arlt *et al*., 2002a; Stiborová *et al*., 2005b), which is widely found in certain areas of Romania, Croatia, Bosnia, Serbia and Bulgaria along the Danube river basin (Tatu *et al*., 1998; Stiborová *et al*., 2005b; Stefanovic *et al*., 2006). At least 25,000 individuals suffer from BEN or are suspected of having the disease, while the total number of people at risk in these countries may exceed 100,000. Although first described more than 50 years ago, the etiology of BEN remains unclear and is a matter of debate (Tatu *et al*., 1998; Stefanovic *et al*., 2006). For the last years evidence has accumulated that BEN is an environmental disease. Recent experimental data shows that AA might be one of the most important etiologic factors in BEN and associated urothelial cancer (Arlt *et al*., 2002a; Stefanovic *et al*., 2006; Grollman *et al*., 2007). AA exposure is associated with chronic dietary uptake of seeds of *Aristolochia clematitis* by the population living in BEN regions (Ivic, 1969; Arlt *et al*., 2002a; Hranjec *et al*., 2005).

## Aristolochic Acid-Mediated Renal Injury and Carcinogenesis

The molecular mechanisms for AA-mediated renal injury, and if it is an early stage of the urothelial-specific tumor development, are still matter of debate and need further investigations. In this context, it is noteworthy that a case of AA-induced tumor development without renal injury (Nortier *et al*., 2003) suggests dissociation between AA-mediated nephrotoxicity and carcinogenicity. AA seems to directly cause renal injury through activating mitochondrial permeability transition, which was found recently in human renal tubular epithelial cells (Qi *et al*., 2007). This suggestion, however, needs to be confirmed by further studies. In contrast to suggestion that AA might be the direct cause of the instestitial nephropathy, metabolic activation of AA to species forming DNA adducts is an important step for AA-induced malignant transformation (Arlt *et al*., 2007; Stiborová *et al*., 2008b). Indeed, the molecular mechanism of AA-induced carcinogenesis demonstrates a strong association between DNA adduct formation, mutation pattern and tumour development (Arlt *et al*., 2007). The predominant AA-DNA adduct, 7-(deoxyadenosin-N^6^-yl)aristolactam I (dA-AAI), which is the most persistent of the adducts in the target tissue, is a mutagenic lesion leading to A→T transversions in the *p53* gene in DNA from urothelial tumors of AAN and BEN patients (Lord *et al*., 2004; Arlt *et al*., 2007; Grollman *et al*., 2007).

## Metabolism of Aristolochic Acid and Biotransformation Enzymes

One of the common features of AAN and BEN is that not all individuals exposed to AA suffer from nephropathy and tumor development. We have suggested earlier that one cause for these different responses may be individual differences in the activities of the enzymes catalyzing the biotransformation (detoxication and/or activation) of AA (for a summary, see Stiborová *et al*., 2008b) Many genes of enzymes metabolizing toxicants and carcinogens are known to exist in variant forms or show polymorphisms resulting in differing activities of the gene products. These genetic variations appear to be important determinants of cancer risk or other toxic effects of xenobiotics (Stiborová *et al*., 2008b).

**Figure 2:**
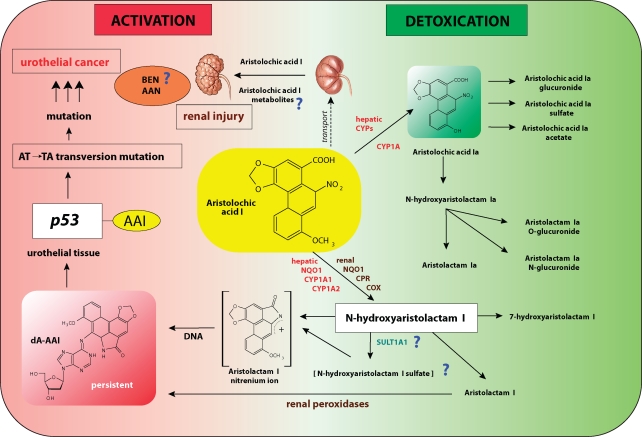
Proposed pathway for metabolic activation and detoxication of aristolochic acid I (AAI), leading to renal injury and urothelial cancer. Aristolochic acid nephropathy (AAN); Balkan endemic nephropathy (BEN); 7-(deoxyadenosin-N6-yl)aristolactam I (dA-AAI); cytochrome P450 (CYP); NADPH:CYP reductase (CPR); cyclooxygenase (COX); sulfotransferase (SULT) (adapted from reference Stiborová *et al*., 2008c).

The proposed activation and detoxication pathways for the major component of AA, aristolochic acid I (AAI), are shown in the Figure 2. AAI is activated by simple nitroreduction to *N*-hydroxyaristolactam I that forms a cyclic *N*-acylnitrenium ion as the ultimate carcinogenic species binding to DNA to form 7-(deoxyadenosin-N^6^-yl)aristolactam I (dA-AAI) as the major persistent adduct, participating in initiation of carcinogenesis. The enzymes activating AA to species binding to DNA *in vitro* were studied in details (Stiborová *et al*., 2008b). Using *in vitro* studies we have found that the most important human and rat enzyme activating AAI *in vitro* in hepatic and renal cytosolic subcellular fractions is NAD(P)H:quinone oxidoreductase (NQO1)(Stiborová *et al*., 2002a; 2003; 2005a) followed by cytochrome P450 (CYP) 1A1/2 in liver microsomes (Stiborová *et al*., 2001c; 2005c) and NADPH:CYP reductase (POR) in kidney microsomes (Stiborová *et al*., 2001b; 2005a), besides prostaglandin H synthase (cyclooxygenase, COX) (Stiborová *et al*., 2001a), which is highly expressed in urothelial tissue. However, the confirmation that isolated (purified) human NQO1 is really capable of activating AAI remains still to be investigated. Such a proof is of great importance. It is namely noteworthy that NQO1-polymorphism (the genotype NQO1*2/*2) seems to predispose patients suffering from BEN to the development of urothelial malignancy of the upper urinary tract (OR=13.75, 95%CI 1.17-166.21) (Toncheva *et al*., 2004). Therefore, the targets of our future work are the confirmation of this finding (predisposition of patients to the development of cancer by NQO1-polymorphism), and the confirmation of the major role NQO1 in AAI activation. The results of such studies might answer the question why AAI-induced cancer is developed in only some of the AAN and BEN patients.

The competing conversion of *N*-hydroxyaristolactam I to the corresponding 7-hydroxyaristolactam or its further reduction to aristolactam I should be considered detoxication pathway; both these metabolites were found to be excreted. However, even though aristolactam I is not a direct DNA binding species, low amounts of the dA-AAI adduct, with the highest levels in one of the target tissues, the renal pelvis, were generated in rats treated with aristolactam I (Dong *et al*., 2006). This result is consistent with finding that formation of the dA-AAI adduct by aristolactam I was observed after its *in vitro* activation with different peroxidases of which several, such as COX-1 and/or COX-2, are expressed at high levels in renal tissue (Stiborová *et al*., 1999). It is still questionable if enzymes capable of conjugating the proximate carcinogenic metabolite of AAI, *N*-hydroxyaristolactam I, are involved in AAI activation. Meinl *et al*. (2006) demonstrated that expression of some human sulfotransferases (SULTs), particularly SULT1A1, in bacterial and mammalian target cells enhances the mutagenic activity of AAI. However, our preliminary experiments did not bring unambiguous results. On one hand, we found that an increase in AAI-induced mutagenicity was correlated with higher AAI-DNA adduct levels in V79 cells transfected with human SULT1A1 (Glatt *et al*., unpublished results). However, our further results suggest that SULTs in human hepatic and renal cytosols do not participate in an increase in AAI-DNA adduct formation in these subcellular systems. Thus, the exact role of conjugation enzymes in AAI activation awaits further investigation and is another aim of our additional studies.

While most of the enzymes catalyzing the reductive activation of AAI *in vitro* have already been identified, the question, which of them actually participates in this process *in vivo*, remains to be answered. Additional factors such as route of administration, absorption, renal clearance and tissue-specific enzyme expression make it difficult to extrapolate from data found *in vitro* (Stiborová *et al*., 2008b) to the *in vivo* situation. Such a study is, therefore, the next target of our future investigation.

The oxidation of AAI to aristolochic acid Ia (AAIa) has been suggested to be a detoxication pathway of AAI (Arlt *et al*., 2002b; Stiborová *et al*., 2008b). Namely, AAIa or its conjugates, the *O*-glucuronide, the *O*-acetate and the *O*–sulfate esters, were excreted in urine (Chan *et al*., 2006; 2007). AAIa is also reduced to *N*-hydroxyaristolactam Ia forming aristolactam Ia, which together with its conjugates, the *N*- and *O*-glucuronides, is excreted (Chan *et al*., 2006; 2007). Enzymatic reactions leading to aristolactam Ia and its metabolites seem to be purely detoxication pathway, because DNA adducts containing aristolactam Ia structure have as yet never been found. In contrast to the enzymes activating AAI *in vitro*, those participating in AAI oxidation to AAIa both *in vitro* and *in vivo* have not been extensively studied so far. Our preliminary studies indicated that CYP enzymes can generate this oxidative metabolite (Stiborová *et al*., 2008b). However, the question which of the CYP enzymes are responsible for formation of AAIa remains still to be investigated. In this context it is noteworthy that a large-scale investigation in BEN patients on the role of genetic polymorphisms in genes of some phase I detoxication CYP enzymes revealed a possible risk for BEN (OR 2.41) in individuals carrying CYP3A5*1 allele G6989 (Toncheva *et al*., 2004; Toncheva 2006). Although we found that this CYP did not activate AAI to dA-AAI adduct (Stiborová, unpublished results), we do not know, if this CYP species is involved in AA detoxication. Furthermore, as mentioned above, we also do not know if other CYPs, and which of them, are capable of detoxicating AAI. Therefore, the evaluation of the oxidative detoxication of AAI by individual CYP enzymes is the target of studies in several laboratories. Indeed, very recently, Xiao *et al*. (2008) showed novel data concerning the enzymes detoxicating AAI. The HRN (Hepatic Cytochrome P450 Reductase Null) mice, which we had shown previously to be a suitable model to determine hepatic xenobiotic metabolism *in vivo* (Arlt *et al*., 2005; 2006; 2008; Stiborová *et al*., 2008a) and suggested to use to elucidate AA metabolism (Stiborová *et al*., 2008b), were successfully used in their study (Xiao *et al*., 2008). The authors' results indicate that hepatic CYPs detoxify AAI by its demethylation to aristolochic acid Ia (AAIa), and thereby protect the kidney from AAI-induced injury. The observations of Xiao *et al*. (2008) combined with results found previously, support strongly the former hypothesis (Stiborová *et al*., 2008b) that a key point determining the carcinogenic and nephrotoxic effects of AAI lies in the balance of activities of reductases such as NQO1, catalyzing the AAI-DNA adduct formation, and enzymes such as CYPs, which detoxicate AAI to AAIa.

The question which of the CYP enzymes are responsible for formation of AAIa remains still to be investigated. The *in vitro* experiments of Xiao *et al*. (2008) indicate that CYP1A generate AAIa. However, the model used to evaluate CYP1A participation in formation of AAIa *in vivo*, mice treated with an inducer of CYP1A 3-methycholanthrene (MC), did not bring unambiguous results. Namely, MC also induces other enzymes beside CYP1A. Although treatment of mice with MC leads to decrease in AAI concentrations in the liver and kidney, no increase in AAIa concentrations was found in the liver, only in the kidney of mice treated with the higher dose of AAI (20 mg/kg). An increase in AAIa excretion due to its conjugation with glucuronide, caused by induction of UDP-glucuronosyltransferase with MC, could occur. Nevertheless, because CYP1A enzymes also activate AAI to species forming DNA adducts (Stiborová *et al*., 2008b), the decrease of AAI in liver and kidney might also result from this reaction. Moreover, NQO1, which is also efficiently induced by MC, could contribute to decreased AAI levels in MC-treated mice.

Taking into account all data known at the present time, we propose that the pathways of AAI metabolism are dictated by the binding affinity of AAI to CYP1A or NQO1, and their enzymatic turnover as well as by the balance between the efficiency of CYP1A to oxidize and reduce AAI. In order to confirm this assumption and to complement our former studies (Stiborová *et al*., 2008b; c), and the work of Xiao *et al*. (2008), we started a study investigating formation of AAI-DNA adducts in the HRN mouse model, and in models, in which *CYP1A* genes are deleted.

## Conclusions

Although hepatic CYP enzymes were found to detoxicate AAI in mice, thus decreasing its renal toxicity (Xiao *et al*., 2008), individual enzymes, which might metabolize (activate and/or detoxicate) AAI *in vivo*, and their impact on AAI-mediated nephrotoxicity and carcinogenicity, have not been fully resolved as yet. Therefore, such a subject remains to be investigated. Namely, the evaluation of inter-individual variations in the human enzymes playing a major role in AAI activation and detoxication, including their genetic polymorphisms, remain a major challenge to explain an individual's susceptibility to AAI, and to predict cancer risk among the AAN and BEN patients. Therefore, the study we started in our laboratory addresses still unsettled question whether the metabolism of AAI, and if so, which enzymes participating in this process, determine pathophysiological effects of this compound in development of AAN and BEN diseases.
